# Kyste hydatique utérin: localisation rare

**DOI:** 10.11604/pamj.2021.39.272.29631

**Published:** 2021-08-26

**Authors:** Issam Loukil, Amine Zouari

**Affiliations:** 1 Service de Chirurgie Générale Tataouine, Tataouine, Tunisie,; 2 Service de Chirurgie Générale Sfax, Sfax, Tunisie

**Keywords:** Hydatidose, kyste, utérus, Hydatidosis, cyst, uterus

## Abstract

We here report the case of B.J, an 83-year-old patient with no previous history, presenting with intermittent abdominal pain evolving over the last few months. Physical examination revealed the presence of a firm, subumbilical mass that was tender to palpation. Ultrasound showed two multivesicular hydatid cysts (Gharbi’s classification type 3) located in segments III and IV of the liver and multilocular right latero-uterine cyst. Serologic test was positive. Tumor markers were negative. Abdomino-pelvic computed tomography (CT) scan showed two adjacent multivesicular hydatid cysts in the left side of the liver measuring 60x40 mm (A), and a multivesicular right latero-uterine pelvic hydatid cyst measuring 110x80 mm pushing the bladder forward and the uterus to the left (B). Surgical exploration revealed the presence of left hepatic cysts (C) and a huge hydatid cyst arising from the right edge of the uterine body (D). Resection of the salient domes was performed. All the precautionary measures were implemented to avoid possible peritoneal dissemination. Anatomopathological examination confirmed the diagnosis of hydatid cysts. One-year CT scan did not show any local or peritoneal recurrence.

## Image en médecine

Patiente B.J âgée de 83 ans, sans antécédents, consulte pour des douleurs abdominales intermittentes évoluant depuis quelques mois. L´examen physique note la présence d´une masse abdominale sous ombilicale ferme et sensible à la palpation. Une exploration échographique a montré la présence de deux kystes hydatiques multi vésiculaires des segments III et IV du foie, type III selon la classification de Gharbi et un kyste latéro-utérin droit multi loculé. La sérologie hydatique est positive. Les marqueurs tumoraux sont négatifs. Le scanner abdomino-pelvien a montré deux kystes hydatiques contigus du foie gauche multi vésiculaires de 60x40 mm (A), et un kyste hydatique pelvien latéro-utérin droit multivésiculaire de 110x80 mm refoulant la vessie en avant et l´utérus à gauche (B). L´exploration chirurgicale a mis en évidence les kystes du foie gauche (C) et un énorme kyste hydatique prenant naissance sur le bord droit du corps utérin (D). L´intervention chirurgicale par résection des dômes saillants a été réalisée avec toutes les mesures de précaution pour éviter une éventuelle dissémination péritonéale.

**Figure 1:**
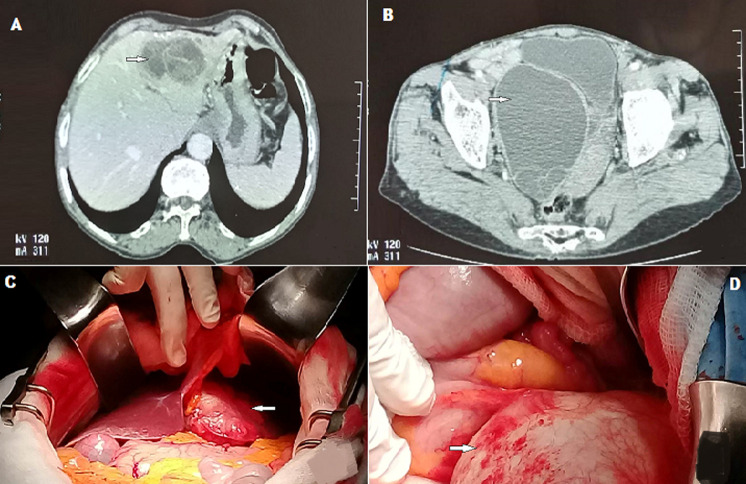
tomodensitométrie (TDM) abdomino-pelvien: A) deux kystes hydatiques contigus du foie gauche multivésiculaires de 60x40 mm; B) un kyste hydatique pelvien latéro-utérin droit multivésiculaire de 110x80 mm refoulant la vessie en avant et l’utérus à gauche; C) kystes du foie gauche; D) énorme kyste hydatique prenant naissance sur le bord droit du corps utérin

